# Development of multivariable models to predict change in Body Mass Index within a clinical trial population of psychotic individuals

**DOI:** 10.1038/s41598-017-15137-7

**Published:** 2017-11-07

**Authors:** Rebecca N. S. Harrison, Fiona Gaughran, Robin M. Murray, Sang Hyuck Lee, Jose Paya Cano, David Dempster, Charles J. Curtis, Danai Dima, Hamel Patel, Simone de Jong, Gerome Breen

**Affiliations:** 1 MRC Social Genetic and Developmental Psychiatry Centre, Institute of Psychiatry, Psychology and Neuroscience, King’s College London, London, UK; 2 Department of Psychosis Studies, Institute of Psychiatry, Psychology and Neuroscience, King’s College London, London, UK; 3 NIHR Biomedical Research Centre at South London and Maudsley NHS Foundation Trust and King’s College London, London, UK; 4 Department of Psychology, City, University of London, London, UK; 5 Department of Neuroimaging, Institute of Psychiatry, Psychology and Neuroscience, King’s College London, London, UK; 6 Department of Biostatistics and Health Informatics, Institute of Psychiatry, Psychology and Neuroscience, King’s College London, London, UK

## Abstract

Many antipsychotics promote weight gain, which can lead to non-compliance and relapse of psychosis. By developing models that accurately identify individuals at greater risk of weight gain, clinicians can make informed treatment decisions and target intervention measures. We examined clinical, genetic and expression data for 284 individuals with psychosis derived from a previously published randomised controlled trial (IMPACT). These data were used to develop regression and classification models predicting change in Body Mass Index (BMI) over one year. Clinical predictors included demographics, anthropometrics, cardiac and blood measures, diet and exercise, physical and mental health, medication and BMI outcome measures. We included genetic polygenic risk scores (PRS) for schizophrenia, bipolar disorder, BMI, waist-hip-ratio, insulin resistance and height, as well as gene co-expression modules generated by Weighted Gene Co-expression Network Analysis (WGCNA). The best performing predictive models for BMI and BMI gain after one year used clinical data only, which suggests expression and genetic data do not improve prediction in this cohort.

## Introduction

Psychosis is present in a number of disorders, including schizophrenia and psychotic subtypes of bipolar disorder and depression. Psychosis is characterised by delusions, hallucinations, disorganized thinking and behaviour. For all psychotic disorders, the lifetime prevalence estimate is 3.06%^1^ and the lifetime incidence is 31.7 per 100,000 person years^2^.

Psychosis is primarily treated by antipsychotics, occasionally supplemented with additional psychotropic medications. Among antipsychotics, clozapine and olanzapine are associated with the greatest weight promoting effects^3,4^. Weight gain has been shown to be a ‘very likely’ cause in patient non-adherence to medication^5^, which is estimated at 41% among individuals with schizophrenia^6^. Non-adherence increases risk of psychotic relapse, and each psychotic episode may contribute to treatment resistance and worse outcome^7^. Psychiatric patients tend to also have lower levels of physical health, and are more likely to suffer complications relating to obesity^8^. A model capable of accurately identifying individuals at risk of weight gain would allow limited weight intervention resources to be targeted effectively, and indicate to clinicians that a less weight-promoting antipsychotic or weight loss medications could be beneficial.

Individuals receiving the same medication display extensive variation in weight gain. This variation may be due to environmental, demographic, genetic or expression differences. Clinical and demographic variables have been shown to influence weight gain risk in individuals receiving antipsychotics. In a study of 65 patients on clozapine, olanzapine or risperidone over an average of 7 years, females were more likely to gain weight^9^. Similarly, psychotic individuals have a greater risk of changing from normal BMI to overweight or obese BMI than the general population, with females having a 3.6 fold risk and males a 2.1 fold risk^10^. Younger age is associated with antipsychotic induced weight gain^9,11^, as is black and African American ancestry^12^. Co-prescription of other weight promoting drugs such as antidepressants, mood stabilizers, anti-histamines, beta blockers and steroids have been associated with weight gain in a meta-analysis of individuals over 19 years of age^13^.

It has been shown that certain single nucleotide polymorphisms (SNPs) in weight -associated genes such as FTO, LEPTIN and MC4R pre-dispose individuals to developing antipsychotic-induced weight gain^14–16^. Polygenic risk scores (PRS) combine the odds ratios of significant and sub-threshold SNP genotypes to calculate an individual’s ‘genetic burden’ for weight gain^17^. Polygenic risk scores of 32 SNPs have been associated with BMI and shown to improve obesity prediction in patients with major depressive disorder (MDD)^18^. Similarly, a genetic risk score of 56 SNPs was associated with BMI in the Molecular Genetics of Schizophrenia controls after controlling for ancestry, sex and age^19^.

Individuals with increased body mass index (BMI) have been reported to display different gene expression patterns relative to those of normal BMI. In a study of omental adipose tissue from five obese and six non-obese pre-pubescent children, 342 differentially expressed genes were found between groups^20,21^. Similarly, extensive weight loss after bariatric surgery leads to significant increased expression in adipose tissue of several genes involved in lipid and mitochondrial metabolism^22^.

Psychotropic mediations can induce gene expression changes. A separate study focussing on clozapine within the same cohort found no large differences in whole blood gene expression between groups of individuals receiving antipsychotics, but clozapine monotherapy induced nominally significant changes in gene expression^23^. In another study of whole blood from 121 schizophrenia patients (92 medicated and 29 unmedicated) and 118 healthy controls, only two modules associated with schizophrenia in medicated individuals were replicated in unmedicated schizophrenia patients, suggesting some expression changes associated with schizophrenia could be due to the influence of medication^24^.

This study describes the development, selection and internal validation of a machine-learning model to predict BMI change in individuals with psychosis. Regression models predicted final BMI and classification models predicted occurrence of BMI gain. Models were built on combined clinical, genetic and expression data (n = 108), clinical and genetic data (n = 108), clinical and expression data (n = 108), clinical data (n = 108), and finally all available clinical data (n = 284).

## Methods

### Ethical approval

Ethical approval was obtained from The Joint South London and Maudsley and The Institute of Psychiatry NHS Research Ethics Committee (REC ref no. 09/H080/41). All participants gave informed consent and all experiments and methods were conducted in accordance with the relevant guidelines and regulations, including Consolidated Standards of Reporting Trials (CONSORT) cluster trial extension standards^25,26^, and Transparent reporting of a multivariable prediction model for individual prognosis or diagnosis (TRIPOD)^27^.

### Data

The data originated from a previously published randomised controlled trial named Improving physical health and reducing substance use in Psychosis (IMPACT)^28^ Patients had a diagnosis of a psychotic disorder (ICD 10 diagnosis F20-29, F31.2, F31.5) and were 18–65 years old. (See supplementary methods). Descriptive characteristics are shown in Table 1. Out of 406 patients, 284 individuals had Body Mass Index (BMI) measurements at baseline and after one year, following removal of individuals with extreme baseline BMI (over 55 kg/m^2^, n = 3). Regression models assessed BMI after one year as a continuous variable. Classification models predicted BMI gain as a binary variable, defined as a BMI point increase of ≥1 relative to baseline. This clinical dataset was analysed in two strata. The larger dataset (n = 284) included all individuals with clinical and BMI change data at 1 year. The smaller dataset comprised of individuals with additional genetic and expression data available.

**Table 1:** Demographic and clinical statistics for both datasets. ±indicates standard deviation of the means.

Variable	N = 108	N = 284
Age	45.36 ± 9.25	44.92 ± 9.9
%Male	62.9%	59.9%
Mean baseline BMI	31.63 ± 7.16	30.98 ± 6.92
Mean PANSS	48.78 ± 11.19	50.68 ± 13.12
% smokers	62.0%	62.6%
Mean number of weight promoting drugs	2.79	2.14
**Diagnosis**		
Schizophrenia	72	190
Schizotypal	1	1
Delusional	0	3
Schizoaffective	13	44
General psychosis	2	3
Bipolar disorder (with psychosis)	16	32
Depression	4	11
**Ethnicity**		
White	57	159
Black Caribbean	29	67
Black African	8	24
Asian	7	11
Mixed/Other	7	23

### Data pre-processing

**Figure 1:**
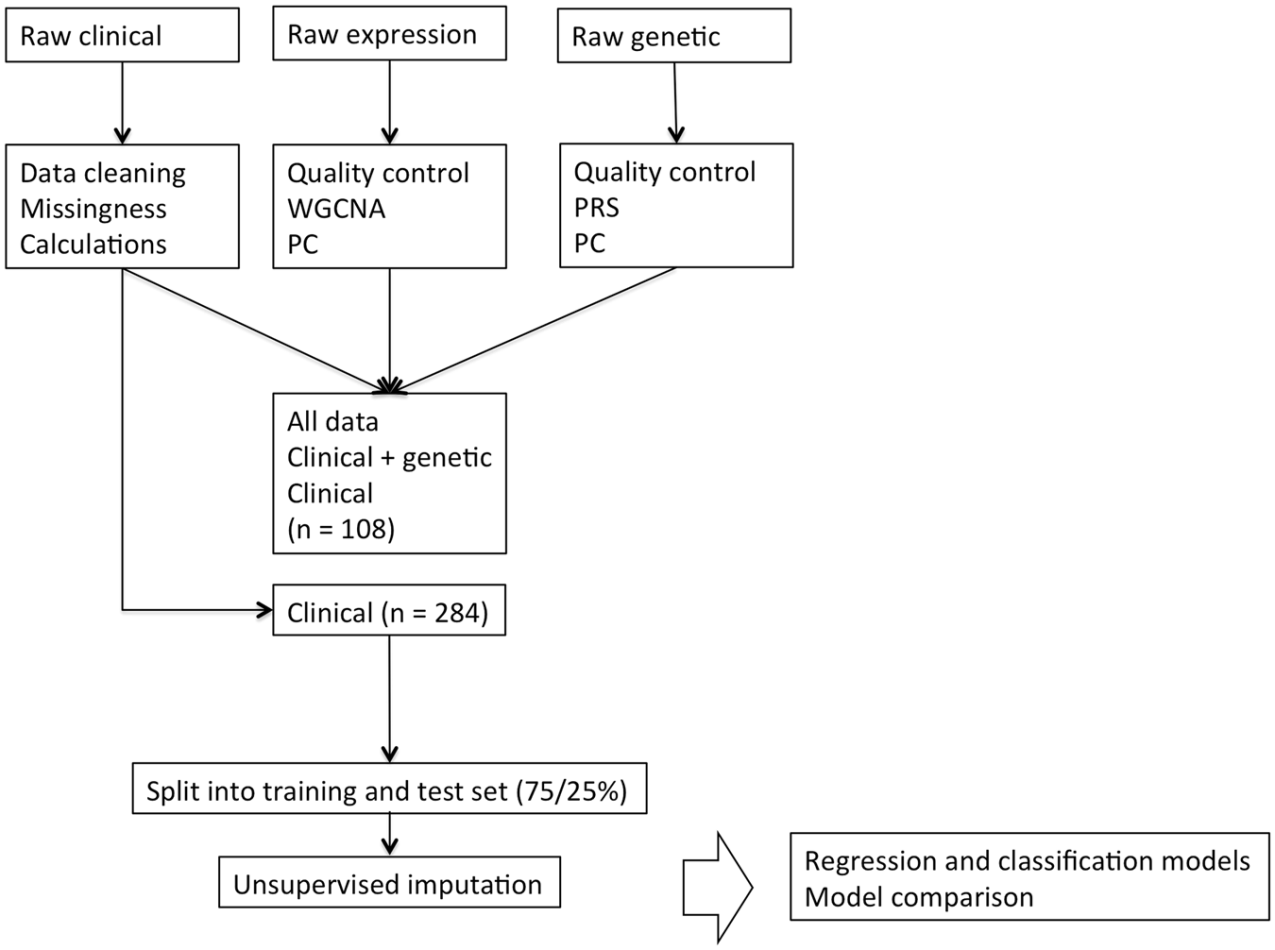
Data processing steps and model development. PRS, polygenic risk score; PC, principal components; WGCNA, weighted gene correlation network analysis.

The general pipeline is outlined in Fig. 1. A summary of the variables used in the final model is shown in Supplementary Table 1.

### Clinical

Clinical data with missingness of less than 35% for 54 variables was imputed using K-fold nearest neighbour (KNN)^29^. Samples or variables with higher missingness were excluded. Missing values were calculated via weighted averages of the Euclidian distance between the ten most similar complete-case ‘donors’ and the sample with a missing value. Training and testing data imputation was separate and unsupervised to reduce bias.

Medication was categorized as weight-promoting according to the British National Formulary (BNF)^30^. A drug categorized as weight promoting had weight gain or weight changes as a very common, common, uncommon or rare side effect. A drug was also defined as weight promoting if the risk of weight gain was higher than weight loss. The medications defined as weight promoting are shown in Supplementary Table 2. In the full cohort of 284 individuals, there were 270 individuals who received at least one weight-promoting drug. The mean number of weight promoting drugs per individual was 2.14. In the subset of 108 individuals there were 105 who received at least one weight-promoting drug. The mean number of weight promoting drugs prescribed was 2.79.

### Genetic

The Infinium CoreExome array (Illumina, California) generated genetic data for 551,839 markers. Quality control followed an established pipeline^31^. 249 individuals and 293,704 variants passed quality control. The iterative filtering thresholds used were minor allele frequency (MAF) = 0.01 and Hardy Weinberg Equilibrium (HWE) = 0.00001. Total genotyping rate was 0.99. Polygenic risk scores (PRS) for schizophrenia^32^, bipolar disorder^33^, BMI^34^, waist-hip-ratio^34^, insulin resistance^35^ and height^36^ were generated using PRSice^37^, including clumping, with threshold ranges from 0 to 0.5 with 0.01 increments. The selected threshold for each risk score and the number of SNPs is shown in Supplementary Table 3.

Principal components (PCs) were generated utilizing Principal Component Analysis (PCA) from linkage disequilibrium pruned data, which included 249 individuals and 93,265 variants. Genetic principal components were used to highlight any hidden effects of ancestry. The correlations of 63 clinical, PRS and celltype variables with genetic PCs were assessed, and significant correlations are noted in Supplementary Table 4 (p < 0.05/63 = 7.9 × 10^−4^). We included the genetic principal components in addition to the correlated variables identified in Supplementary Table 4 to maximise the choice of variables to the machine learning algorithms and to see if principal components would be chosen as a proxy summary measure in place of several correlated variables.

### Expression

HumanHT-12.v4 BeadChips (Illumina, California) generated expression data for 391 samples and 47323 probes across three time points. Samples and probes were quality controlled using an in-house pipeline (https://github.com/snewhouse/BRC_MH_Bioinformatics). 376 samples had complete technical, sample and probe information. 14 samples were removed based on network similarity, assessed with Weighted Gene Co-expression Network Analysis (WGCNA)^21^. Background correction of probes compared expression against the negative control data using Maximum Likelihood Estimation (MLE). 6,359 high quality probes were detected across all time points. Non-baseline individuals were removed, resulting in 6,359 probes and 153 individuals. 40 sex fails identified based on XIST expression were removed, leaving 108 individuals with BMI data, baseline expression data and genetic data (Supplementary Figure 1).

The CellMix package within R^38^ with a pre-determined list of blood cell type markers^39^ estimated enrichment for lymphocytes, monocytes and neutrophils. Principal components within the expression data were generated. The BMI expression data was corrected for expression principal components PC1, PC2, PC7, and PC8. PC1 accounted for 23.78% of the variance in expression data and was significantly associated with 12 variables, including ethnicity, schizophrenia PRS, Height PRS, waist circumference, PC1 genetic and cell type. PC2 accounted for 8.15% of the variance and was significantly associated with 10 variables, including technical variables. PC7 accounted for 2.81% of the variance and was significantly associated with 29 variables, including cell type (monocytes) and batch effect technical variables. PC8 accounted for 2.69% of the variance and was significantly associated with 51 variables, including many technical variables. The association of significant clinical variables, cell types, genetic PRS and genetic PCs with expression PCs is shown in Supplementary Table 5 (p < 0.05/73 = 6.8e-4).

The expression data was corrected for these principal components utilizing a linear model in R. The residuals were used to generate twelve modules via Weighted Gene Co-expression Network Analysis (WGCNA)^21^. Residuals were used to adjust for large variations in the data prior to module generation within a single network. WGCNA is a systems biology method used to analyse microarray expression data as a network. Genes are clustered relative to their shared neighbours and assigned to a module. The module eigengene of each module represents the first principal component of the corrected expression values within each module. This reduces the number of tests performed. An unsigned network allowed for multi-directional effects, and a soft power threshold of 4 exceeded the 0.9 R^2^ value to prioritise highly connected genes within modules. The modules significantly associated with clinical and genetic variables are shown in Supplementary Table 6. The AmiGO tool^40^ (version 2.5) with PANTHER^41^ (version 12.0) and Gene Ontology Database (released 14/08/2017) was used to assess enrichment of each module, using the PANTHER Overrepresentation Test (release 13/04/2017) with all probes detected in the full dataset as the background reference. We tested the all annotation datasets available; PANTHER Pathways, PANTHER GO-Slim Molecular function, PANTHER GO-Slim biological process, PANTHER GO-Slim Cellular component, PANTHER protein class, GO cellular component, GO molecular function, GO biological process and Reactome Pathways.. If significant enrichment was given as ‘Unclassified’, we reported no enrichment for that module. For simplicity, we report the main enrichment category rather than category subsets. We also provide a list of genes within each module, generated by matching Entrez IDs with Ensembl gene identifiers using Database for Annotation, Visualization and Integrated Discovery (DAVID) v6.8^42,43^.

### Model generation

Eleven standard regression methods and ten classification methods were tested in an empirical machine learning approach. Models were developed in a ‘training’ subset (75%) and tested in a ‘testing’ subset (25%). The allocation of individuals to these subsets was done utilising the createDataPartition function in the caret package^44^, with a seed for reproducibility. The demographics of the training and testing data are shown in Supplementary Table 7. Machine learning selects and evaluates predictors based on their ability to predict the outcome rather than with any prior biological association^45^. This was done within the caret package in R^46^. The model methods used for both regression and classification included classification and regression trees (CART), random forests^47^, bagging^48^, Generalised linear models with elastic and lasso net regularisation (utilising the Generalised Linear Model package)^49^, linear support vector machines (SVM)^50^, K-nearest neighbours(KNN)^51^ and generalized boosting model (GBM)^52^. Methods used for regression included ridge regression^53^, boosted linear regression^54^, elastic net^55^, Independent Component Regression (ICR)^56^. Methods specific to classification were polynomial and radial SVM and classification trees based on Quinlan’s C5.0 algorithm^57^. All models were generated with a non-random seed for reproducibility.

We focus on the methods that featured prominently in the best models. Generalised linear models via the package glmnet^49^ fit a model via a penalized maximum likelihood framework. The α penalty varies between 0 (ridge regression) and 1 (lasso regression). Lambda indicates the penalty strength. KNN (k-nearest neighbours) is a non-parametric method that uses data from its most similar neighbours for prediction^51^. Tree based methods such as random forests generate many trees tuned by depth and number of predictors considered per node^47^.

### Model assessment

The mean performances of the models in training data was assessed following 10-fold internal cross-validation repeated 10 times. Internal cross-validation avoids over-fitting by building and testing the model on unseen data from the same dataset. The performance confidence intervals in the training data were calculated from the overall standard deviation across all folds.

The best performing model was decided based on performance in the training set (75%), considering ease of interpretability if performance was very similar ( ±0.1 for RMSE or kappa, ±0.01 for R^2^ and accuracy). These models were tested in the testing data (25%) and the performance in the test set was reported. Models classifying all individuals in one class were discounted. Performance of classification models was evaluated using mean accuracy, kappa, specificity, sensitivity, positive predictive value (PPV) and negative predictive value (NPV). Performance of regression models was assessed using root mean square error (RMSE) and R^2^. The performance of each dataset was then ranked according to performance in testing data. These are defined below in equations (1) to (8):

1 $$Accuracy=\frac{number\,of\,correct\,predictions}{total\,number\,of\,predictions}$$

2 $$Kappa=1-\frac{1-observed\,probability}{1-chance\,probability}$$

3 $$Positive\,predictive\,value=\frac{number\,of\,true\,positives}{total\,number\,of\,positive\,predictions}$$

4 $$Negative\,predictive\,value=\frac{number\,of\,true\,negatives}{total\,number\,of\,negative\,predictions}$$

5 $$Sensitivity=\frac{number\,of\,true\,positives}{number\,of\,true\,positives+number\,of\,false\,negatives}$$

6 $$Specificity=\frac{number\,of\,true\,negatives}{number\,of\,true\,negatives+number\,of\,false\,positives}$$

7 $${R}^{2}=\,1-\frac{\sum {(observedBMI-predictedBMI)}^{2}}{\sum observed\,BMI-mean\,{(observedBMI)}^{2}}$$

8 $$RMSE=\sqrt{\frac{{(observedBMI-predictedBMI)}^{2}}{N}}$$

We assessed variable importance utilising the VarImp function within the caret train package^44^. This calculates importance from the ranked coefficients, adjusted for the number of variables within the model. Here, we discuss the top five most important variables for each model. The top 20 most important variables are presented in Supplementary Tables 8 and 9.

### Data availability

The datasets analysed for the current study are available from the corresponding author on reasonable request.

## Results

The best performing model was selected based upon ten-fold repeated cross-validation of the training data. The performance of these ‘best’ regression and classification models in the test data for each data set is displayed in Tables 2 and 3 respectively. The five datasets tested were; Model A built on combined clinical, genetic and expression data (n = 108), Model B with clinical and genetic data (n = 108), Model C with clinical data (n = 108), Model D with clinical and expression data (n = 108), and finally all available clinical data (n = 284). Detailed results for each trialled model per dataset are displayed in Supplementary Tables 10 and 11. We also tested models with only genetic data and only expression data, which are given in Supplementary Tables 12 and 13. Performance in these models was generally worse than the models presented below.

**Table 2:** Model performance in training and testing data for the best regression models in each dataset.

Model	Method	Train R2	Train RMSE	Test correlation	Test R^2^	Test RMSE	Tuning parameter	Rank
E-Clinical (N = 284)	Generalised Linear Model	0.782 [95% CI: 0.769–0.796]	3.51 [95% CI: 3.40–3.62]	0.919	0.829	2.84	alpha = 0.55 and lambda = 1.25	1
C-Clinical N = 108	Generalised Linear Model	0.832 [95% CI: 0.810–0.853]	3.43 [95% CI: 3.20–3.66]	0.900	0.796	2.98	alpha = 0.55 and lambda = 1.32	2
B-Clinical, genetic (N = 108)	Generalised Linear Model	0.830 [95% CI: 0.808–0.851]	3.45 [95% CI: 3.22–3.67]	0.900	0.796	2.98	alpha = 0.55 and lambda = 1.32	=2
D-Clinical, Expression (N = 108)	Generalised Linear Model	0.829 [95% CI: 0.807–0.851]	3.46 [95% CI: 3.24–3.69]	0.896	0.788	3.04	alpha = 0.55 and lambda = 1.32	3
A-Clinical, genetic, Expression (N = 108)	Generalised Linear Model	0.827 [95% CI: 0.805–0.849]	3.48 [95% CI: 3.25–3.71]	0.896	0.788	3.04	alpha = 0.55 and lambda = 1.32	=3

RMSE = root mean squared error, R2 = R-squared, correlation indicates the agreement between predicted and actual values for the test data. CI = confidence interval (95%).

**Table 3:** Model performance in training and testing data for the best selected classification models for each dataset. PPV = Positive predictive value. NPV = Negative predictive value. CI = confidence interval (95%).

Model	Method	Train accuracy	Train Kappa	Test Accuracy	Test Kappa	Test Sensitivity	Test Specificity	Test PPV	Test NPV	Tuning parameter	Rank
E-Clinical (N = 284) First Model	Random Forest	0.608[95% CI = 0.598–0.618]	0.052 [95% CI = 0.028–0.075]	0.586	−0.022	0.61	0.33	0.91	0.07	Mtry = 2	1
E-Clinical (N = 284) Second Model	Generalised Linear model	0.574 [95% CI = 0.561–0.587]	0.056[95% CI = 0.028–0.083]	0.600	0.132	0.66	0.48	0.72	0.41	Alpha = 0.1, lambda = 0.019	(1)
A-Clinical, genetic, Expression (N = 108)	K-Nearest Neighbours (KNN)	0.591 [95% CI = 0.556–0.625]	0.096 [95% CI = 0.022–0.170]	0.577	0.077	0.60	0.50	0.80	0.27	K = 9	= 2
B-Clinical, genetic (N = 108)	KNN	0.591 [95% CI = 0.556–0.625]	0.096 [95% CI = 0.022–0.170]	0.577	0.077	0.60	0.50	0.80	0.27	K = 9	= 2
D-Clinical, Expression (N = 108)	KNN	0.591 [95% CI = 0.556–0.625]	0.096 [95% CI = 0.022–0.170]	0.577	0.077	0.60	0.50	0.80	0.27	K = 9	= 2
C-Clinical N = 108	KNN	0.591 [95% CI = 0.556–0.625]	0.096 [95% CI = 0.022–0.170]	0.577	0.077	0.60	0.50	0.80	0.27	K = 9	= 2

A total of 6359 probes for 108 individuals were used in the expression data, which were grouped into a total of 12 modules. This includes 1680 probes within the grey module, generally indicative of noise. The other expression modules ranged in size from 41 probes to 1280. The first 10 genetic principal components explained 32% of the genetic variance. The first 10 expression principal components explained 60.67% of the expression variance, although these were not included in the models because module eigengenes are an equivalent method of proportioning variance. Significant enrichment of pathways for each module is shown in Supplementary Table 14.

### Model A: Clinical + Genetic + Expression (n = 108)

Model A utilised clinical data, six genetic polygenic risk scores, ten genetic principal components and twelve expression module eigengenes for 108 individuals. 75% of the data (n = 82) comprised the training set and 25% comprised the testing set (n = 26). In the training data, 47 individuals (43.5%) had BMI gain. The mean BMI after 1 year in training data was 31.65. The demographics are shown in Table 1. The correlation between BMI PRS and BMI measures at one year was not significant, but there was a slight correlation between BMI PRS and weight at 1 year (p = 0.057, correlation = 0.18).

The best method for regression model A in the training data was Generalised Linear Model (RMSE = 3.48; R2 = 0.83). This was the joint third best performing regression model with regression model D (GLM). In testing data for regression model A (GLM), predicted and observed values had a correlation of 0.896, and RMSE = 3.04, R2 = 0.788. The chosen model parameters were alpha = 0.55 and lambda = 1.32, indicating an intermediate ridge- lasso regression parameter, with a reasonable penalty. The coefficients of regression model A (GLM) are shown in Table 4.

**Table 4:** Coefficients of generalised linear models.

	Reg. A	Reg. B	Reg. C	Reg. D	Reg. E	Class. E
(Intercept)	−6.870	−7.692	−7.707	−6.886	2.131	−1.377
Diastolic BP	0.017	0.019	0.019	0.017		−0.001
Waist	0.053	0.056	0.056	0.053	0.072	0.022
Hip	0.178	0.183	0.183	0.179	0.048	0.025
Height	.	.	.	.	−0.006	−0.013
BMI	0.348	0.343	0.343	0.348	0.544	−0.060
Fried food	−0.082	−0.098	−0.099	−0.082		−0.214
PC10 genetic	0.103	0.0989	.	.		.
ME pink	2.296	.	.	2.294		.
Intervention status						0.353
Age						0.002
ICD10 diagnosis						−0.007
Borough						0.077
Place of birth						0.081
Ethnicity						0.007
Ethnicity group						0.196
Systolic BP						0.014
Weight						−0.019
Fasting glucose						−0.079
HDL						0.241
LDL						0.009
Triglycerides						0.050
HBA1C percentage						0.156
HBA1C result						−0.032
PCS						−0.025
MCS						0.011
MDRAS total						0.021
PANSS positive						−0.068
PANSS negative						0.009
PANSS GPP						0.034
GAF range						0.154
Walk (hrs)						0.002
IPAQ (walk)						−0.016
Diet- Fat added to Bread/veg						−0.146
Diet- Fat added to Baking						0.113
Diet-sugar						−0.018
Diet-cereal						−0.055
Total fibre						−0.004
Total saturated fat						−0.007
Fibre (category)						−0.047
Total unsaturated fat						−0.004
unsaturated fat (category)						−0.148
Smoker YN						0.866
Cigarettes per day						−0.024
Sex						−0.344
WHR						0.150
Number of weight gain drugs						0.162

Regression model A (GLM) was simple, only including 8 variables. The clinical variables chosen were BMI, hip circumference, waist circumference, weight, diastolic blood pressure and fried food intake. The genetic and expression variables chosen were PC10 and the pink expression module. The pink module contained 166 probes. There was some significant enrichment after Bonferroni correction for ‘external side of plasma membrane’ (p = 0.0091, 4.14 fold enrichment), signal transduction (p = 0.024, 1.87 fold enrichment), and ‘intracellular organelles’ (p = 0.049, 0.76 fold enrichment) (Supplementary Table 14). In addition, the pink module is significantly correlated with hip circumference measurement (Supplementary Table 6).

The classification model A was the joint second best performing model along with classification models B, C and D. Its performance in training data was significant, as the confidence intervals did not overlap 50%. The accuracy was 0.591 [95% CI = 0.556–0.625], and Kappa was 0.096 [95% CI = 0.022–0.170]. Classification model A used the KNN method, which utilises data from its nine most similar ‘neighbours’ to predict outcomes. Classification model A had slightly reduced performance in testing data (Accuracy = 0.577). Classification model A (KNN) was complex, with over 20 variables. The five most important variables were the clinical variables of HBA1C (percentage and absolute values) and fasting glucose. HBA1C is a measure of glycated haemoglobin, which assesses long-term blood sugar levels. The genetic variables selected were PC9 and PC10. There was no significant correlation of variables with PC9 or PC10, so it is likely that selection of this variable reflects confounding variation. The green-yellow module eigengene was the sixth most important variable. This module was the smallest, with only 41 probes and no significant enrichment was identified after Bonferroni correction. The green-yellow module was significantly correlated with genetic PC1, but genetic PC1 was not included in the model.

### Model B: Clinical + Genetic (n = 108)

Regression model B utilised Generalised Linear Model and was the joint second best performing regression model with Regression model C. In training data for regression model B (GLM), RMSE was 3.45 and R2 was 0.83. In testing data, correlation of predicted and observed was 0.9, RMSE was 2.98 and R2 = 0.796. Regression model B (GLM) selected the same clinical and genetic variables as regression model A (GLM) (BMI, Hip circumference, Fried food intake, Waist circumference, Diastolic blood pressure, Weight, PC10), but in the absence of expression information, higher importance was placed on them. Model performance was marginally better than regression model A (GLM), despite selecting fewer variables.

We added individual variants at the FTO (rs9936385), MC4R (rs12970134) and Leptin receptor (rs12077210, rs12059300) to the models incorporating genetic data and found no improvement in regression models for model A or B.

The performance of classification model B was also identical to classification models A, C and D. Classification model B utilised KNN method, and the top five most important variables were HBA1C (percentage and absolute values), fasting glucose, genetic PC9 and PC10 and physical component score (PCS).

### Model C: Clinical (n = 108)

Regression model C utilised Generalised Linear Model and had equal performance to regression model B (GLM). Regression model C (GLM) chose 6 variables, which were identical to the clinical variables chosen in models A and B (BMI, Hip circumference, Fried food intake, Waist circumference, Diastolic blood pressure and Weight).

Classification model C performed the same as models A, B and D. Classification model C utilised KNN method, and the top five most important variables were HBA1C (percentage and absolute values), fasting glucose, Physical component score and weight.

### Model D: Clinical + Expression (n = 108)

Regression model D utilised Generalised Linear Model and identical performance to model A. Regression model D (GLM) had 7 variables, of which the clinical variables were identical to regression models A–C (BMI, Hip circumference, Fried food intake, Waist circumference, Diastolic blood pressure and Weight), with the addition of the pink expression module discussed above. Classification model D utilised KNN method and performance was identical in training and testing data to classification models A–C. The most important clinical variables were HBA1C (percentage and absolute values), fasting glucose and Physical component score. The expression variable chosen was the green-yellow module, discussed above.

### Model E: Clinical (n = 284)

Model E utilised clinical data for 284 individuals. 109 (38%) had BMI gain. The mean BMI after 1 year was 30.95. This model was used to compare the effects of sample size on model performance, by comparing with model C above which had 108 individuals. Data was spit into a training set (n = 214) and test set (n = 70).

Regression Model E was the best model for regression. The regression method chosen was Generalised Linear Model, based on ease of interpretability, since the random forest and generalised boosting models had similar performance to Generalised Linear Model in training data (see Supplementary Tables 10 and 11). Performance was good in training data(R^2^ = 0.782 [95% CI: 0.769–0.796], RMSE = 3.51 [95% CI: 3.40–3.62]) and in testing data (R^2^ = 0.829, RMSE = 2.84). The correlation between actual and predicted values was 0.919. The parameters were alpha = 0.55 and lambda = 1.25. The most important variables in model E were baseline measures of BMI, waist circumference, hip circumference and height. This was the simplest regression model, with only four variables. The coefficients for this model are shown in Table 4.

We investigated two classification modelling methods for model E. Classification model E (random forest) had significant performance in training data (Accuracy = 0.608[95% CI = 0.618–0.598], Kappa = 0.052 [95% CI = 0.028–0.075]). The accuracy in testing data remained high (0.586) but Kappa decreased to −0.022. This low negative value indicates worse than expected performance of the model, with no agreement between the performance in testing and training data. The most important predictive baseline variables identified for Classification model E (random forest) were Global assessment of functioning, time spent exercising (walking), fasting glucose, and systolic blood pressure. To assess direction of effect, we looked at the correlation of these variables within the full dataset of 284 individuals. There was a positive correlation of BMI with GAF (0.15, p = 0.01), fasting glucose (0.12, p = 0.06) and systolic blood pressure (0.24, p = 4.1^−5^). There was a negative correlation of BMI and hours walked (−0.11, 0 = 0.07). A random forest model is difficult to interpret, as the model is a result of the cumulative information from several classification trees. Given this poor performance and interpretability, we also investigated the Generalised Linear model performance for model E. When compared to selected classification models for A-D, classification model E (GLM) was the worst performing in training data but performed better in testing data. The training data accuracy = 0.574 [95% CI = 0.561–0.587] and kappa = 0.052[95% CI = 0.028–0.083] and performance in testing data remained high (accuracy = 0.60, kappa = 0.13). The parameters of the model were alpha = 0.1 and lambda = 0.019.The most important variables in this complex linear model were smoking status, intervention status, sex, Blood HDL and intake of fried food. The number of weight promoting drugs also featured highly in the model. The coefficients are shown in Table 4.

## Discussion

This study used machine learning to select the best statistical prediction method for five distinct subsets of data from eleven regression and ten classification techniques. These prediction models of BMI utilised combinations of clinical, genetic and expression data. The results of all of these models are included in Supplementary Tables 10–13. These models serve as a comparison to the selected ‘best performing’ models displayed in Tables 2 and 3 for each dataset. The genetic and expression only models had generally poorer performance than the models presented above. This is likely to be due to model instability compared to the models with additional clinical data as these models have very few variables to select, and these variables do not have a strong correlation with BMI.

The primary aim of this study was to identify whether the addition of genetic or expression data improved model performance, while also verifying if regression or classification models were more appropriate for predicting weight gain in a clinical setting.

We found that the model with just clinical data and the largest sample size (n = 284) performed best when compared to the other smaller models (n = 108). This improvement in performance may partially be due to an increase in sample size, but the fact that the clinical model (n = 108) performs equally well as models also incorporating genetic and expression data (n = 108) indicates that the addition of expression and genetic data did not improve model performance. A clinical model may be more useful. Additionally, clinical information is currently cheaper and easier to obtain than genetic and gene expression data given the lack of mainstream pharmacogenomic tests for medication-induced weight gain. Regression and classification models both performed well, but regression models may be more easily interpretable and directly relevant to a patient. The performance of the regression models across the different datasets is similar, which may reflect preferential selection of clinical variables in the models.

The regression clinical model, utilising generalised linear models is easy to interpret given the coefficients in Table 4. The variables associated with an increased BMI are baseline BMI, waist circumference and hip circumference. This suggests individuals with higher baseline BMI are more likely to have a higher BMI after one year. In a study of BMI trajectories in the general population, individuals in all BMI categories gained weight over 18 years, but individuals in higher BMI categories at baseline had higher weight after 18 years^58^. This indicates that individuals with a high BMI are likely to maintain or increase BMI over time. Height comprises part of the measure of BMI, so it is to be expected that height would feature in prediction. In individuals with metabolic syndrome, waist circumference has been shown to be significantly correlated with BMI (R = 0.78, p < 0.01)^59^. It has been suggested that BMI measurements alone may underestimate prevalence of obesity, and that incorporating waist circumference measurements improves estimates of obesity prevalence^60^.

Height had a small negative effect on BMI. This reflects an interesting limitation in the use of BMI. The BMI metric assumes BMI has a strong correlation with weight, but not with height^61^. It has been shown in a study of 25 diverse populations that weight is strongly correlated with BMI. However, in most of the populations studied, it was found that BMI was not independent of height, with a significant difference in the weight-height relationship between males and females^61^. It is possible that taller people have smaller BMI than would otherwise be expected in this cohort.

The best performing classification model for model E utilised the random forest model. The most important predictive baseline variables identified were Global assessment of functioning, time spent exercising (walking), fasting glucose, and systolic blood pressure. These variables have some biological significance, although due to the structure of random forest models, it is difficult to interpret the direction of predictive effect. A higher global assessment of functioning score indicates that the patient has greater social, occupational and psychological functioning. In classification model E (random forest), a higher GAF score is correlated with higher BMI. Individuals with higher fasting glucose measures may be indicative of pre-diabetes, or metabolic syndrome, which is known to be associated with obesity. Increased activity, as measured here by walking time, would be expected to decrease BMI. The systolic blood pressure reading indicates the highest blood pressure when the heart is contracting, and has been shown to increase with increasing BMI in both men and women^62^.

For easier interpretation, we also investigated the generalised linear model for classification of BMI gain. A coefficient model allows inferences to be made about the direction of effect. Interestingly, in a classification model, anthropometric measures of BMI have less of a predictive effect. Variables that have a positive correlation with BMI gain include smoking status, intervention status and high density lipo-protein (HDL) concentration, ethnicity group and number of weight gain drugs prescribed. Variables that have a negative association with BMI gain include dietary variables such as fried food, added fat to diet and sex.

Some of the variables identified above have previous support in other models of BMI. We find that higher BMI is predicted by higher values of fasting glucose. A previous model on predicting weight gain in individuals with Type 2 diabetes used very different variables and methodologies, but found an association with baseline age, HbA1c and sex and weight gain^63^. We also find an association between BMI and number of weight gain drugs prescribed. A recent study predicted early weight gain in individuals starting initial treatment with weight-promoting psychotropic drugs, and found that age and baseline BMI were significantly associated with strong weight gain^64^. In contrast to our results, they found that addition of genetic data (18 genes previously associated with weight gain) improved model performance^64^. We tested SNPs previously associated with BMI, in addition to polygenic risk scores and found that adding candidate genes did not improve model performance relative to PRS alone. This suggests that candidate genes added no more power to the model than using PRS alone. In addition, the sample in the current study had been receiving psychotropic drugs for a period of time before baseline measures were taken, and not all people in the sample were taking weight-promoting drugs. Differences in the time scale and definition of ‘weight gain’ between studies could also account for the discrepancies found^65^. Inclusion of smoking and intervention status reflects the importance of accounting for lifestyle factors and changes.

Some unexpected results include the positive association between HDL and BMI. High BMI been previously associated with low HDL levels^66^. Our finding that fat intake has a negative association with BMI is also unusual, but may be caused by the relatively large time interval between baseline and predicted values. Additionally, in an intervention based trial, individuals with previously unhealthy diets may take measures to improve and hence experience weight loss.

Our study does have some limitations. The small sample size of the models (n = 108) was alleviated in training by use of 10 fold cross validation. However, the use of a single hold-out testing set may limit the estimate of model performance as variance may be increased. Our negative findings regarding prediction by genetic and gene expression data may be explained by a small sample size, as it is possible that genetic polygenic risk scores may be more powerful in larger samples. In addition, the best performing clinical model had a larger sample size, and therefore more power to detect the effect of clinical variables. This difference in power could account for the marginally improved performance of the larger clinical model compared to the equivalent model in the smaller dataset. BMI PRS was not selected as an important feature in the models above which indicates that an accurate measure of BMI is currently more useful than a genetic score or expression profile. Additionally, the use of blood tissue in this study may have been a poor representation of the obesity phenotype under investigation, as other studies have investigated adipose tissue directly.

The use of internal validation to address model over-fitting was necessary given the limitations of the data, but is not a gold-standard approach. The sample originates from south London, which is uniquely ethnically diverse compared to other areas of the United Kingdom. This could have particularly impacted the utility of the polygenic risk scores, as schizophrenia polygenic risk scores have been shown to be most effective in discriminating case-control status in European ancestries^67^. Urban and rural populations may also differ in external factors that influence BMI and psychiatric health, such as diet, availability of convenience foods and density of population. This could limit generalisation of the model outside of London. Treatment intervention is cost effective in the general population^68^, but the psychiatric population may incur additional costs due to non-compliance or psychiatric relapse. Clinicians are also required to balance a patient’s physical health and mental health needs.

Overall, we find that a large sample size of clinical data is most effective at predicting antipsychotic-induced weight gain. Genetic or expression data do not improve model performance in this cohort. However, genetic PRS from specific large studies of antipsychotic-induced weight gain would be useful and may soon be available. Both classification and regression models are useful and perform well, and choice of which model to use would be influenced by its required application. We are hopeful that this study could be used to inform patients of their individual risk weight gain from their medication and indicate if an alternative treatment or targeted health interventions could be useful.

## Electronic supplementary material

Supplementary information

Supplementary File 1
