# Supplementary material for: Development of multivariable models to predict change in Body Mass Index within a clinical trial population of psychotic individuals
Source: Sci Rep. 2017 Nov 7;7:14738. doi: 10.1038/s41598-017-15137-7 (PMC5677086; doi:10.1038/s41598-017-15137-7)
Supplement: Supplementary file 1 — Supplementary information [file 41598_2017_15137_MOESM1_ESM.doc]

Manuscript Title:

Development of multivariable models to predict change in Body Mass Index within a clinical trial population of psychotic individuals

Authors:

Rebecca N.S. Harrison, Fiona Gaughran, Robin M. Murray, Sang Hyuck Lee, Jose Paya Cano, David Dempster, Charles J. Curtis, Danai Dima, Hamel Patel, Simone de Jong, Gerome Breen

Supplementary table 1- A summary of the main variables included within the model selection procedures (BMI - body mass index, HDL - high density lipoprotein, LDL- low density lipoprotein, PANNS - positive and negative syndrome scale for assessment of psychosis, MDRAS-Montgomery-Asberg Depression Rating score, WGCNA- Weighted Gene Co-expression Network Analysis)

| Category | Variable | Definition |
| --- | --- | --- |
| **Outcome variables** | BMI after one year | BMI measurements as a continuous variable |
|  | BMI gain after one year | 0=BMI gain <1  1=BMI gain ≥ 1 |
| **Demographics** | Age | Age in years (from DOB) |
|  | Sex | Female/male |
|  | Ethnicity | 17 groups |
|  | Ethnicity groups | 5 groups (black, white, Asian, mixed, other) |
|  | Place of birth | Y/N UK |
|  | Borough (within London) | Coded borough of residence in London |
| **Cardiac and blood measures** | Diastolic Blood pressure | Blood pressure in arteries at rest |
|  | Systolic Blood pressure | Blood pressure in arteries at contraction |
|  | Cholesterol | Measure of total blood cholesterol after fast (mg/dL) |
|  | Triglycerides | Measure of blood triglycerides after fast (mg/dL) |
|  | Fasting glucose | Glucose measurement (mmols/l) after overnight fast |
|  | Pulse | Beats per minute |
|  | HDL | Measure of blood cholesterol (HDL) after fast (mg/dL) |
|  | LDL | Measure of blood cholesterol (LDL) after fast (mg/dL) |
|  | HBA1C | Glycated haemoglobin for assessment of average blood sugar over time |
| **Anthropometric measures** | Waist circumference | Continuous measure |
|  | Hip circumference | Continuous measure |
|  | Weight | Continuous measure |
|  | Height | Continuous measure |
|  | Baseline BMI | Continuous measure |
|  | Waist-Hip ratio | Ratio of hip to waist circumference |
| **Physical health measures** | Physical component score (PCS) | Measure of physical health and functioning |
|  | Walking (mins) | Minutes of walking per week |
|  | Smoking | Currently smoking (Y/N)  Number of cigarettes per day |
|  | Intervention status | Intervention/Treatment as normal (1/0) |
| **Mental health** | ICD10 diagnosis | ICD10 diagnosis |
|  | Mental component score (MCS) | Assessment of mental functioning |
|  | MDRAS | Continuous depression score |
|  | Global Assessment of Functioning | (GAF- range and score) |
|  | PANNS scores (positive) | Assessment of positive symptoms of psychosis |
|  | PANNS scores (negative) | Assessment of negative symptoms of psychosis |
| **Medication** | Number of weight gain drugs | Number of weight promoting drugs a patient is prescribed |
| **Diet** | Total fibre | Estimate of total fibre consumption per week (continuous and categorical measures (low, medium, high)) |
|  | Total unsaturated fat | Estimate of total unsaturated fat consumption per week (continuous and categorical measures (low, medium, high)) |
|  | Total saturated fat | Estimate of total saturated fat consumption per week (continuous and categorical measures (low, medium, high)) |
|  | Added fat  bread/vegetables  Fried food  Baking | Categorical:  1= Butter/Lard  2= Cooking fat or half-fat butter or half-fat margarine  3= No fat or reduced fat  4= Vegetable oil or spread |
|  | Sugar or fibre cereal | 0= less than 3 times a week  1= 3-5 times a week  2= six or more times a week |
| **Genetic data** | Principal components (10) | The principal components of genetic data |
|  | PRS (6) | Polygenic risk scores for schizophrenia, bipolar, BMI, WHR, insulin resistance and height |
| **Expression** | WGCNA generated modules (12) | Expression modules |

Supplementary table 2- A list of the generic names of weight promoting drugs, identified from British National Formulary (BNF) database

| Drug | Weight effect | Weight loss | Drug Class |
| --- | --- | --- | --- |
| Agomelatine | Rare |  | Antidepressant |
| Amisulpride | Frequency Unknown |  | Antipsychotic |
| Amitriptyline | Frequency Unknown | Frequency Unknown | Antidepressant |
| Amlodipine | uncommon |  | Calcium-channel blockers |
| Aripiprazole | Frequency Unknown |  | Antipsychotic |
| Atazanavir | uncommon |  | Anti-retroviral |
| Desogestrel | uncommon |  | Hormones |
| Citalopram | Frequency Unknown |  | Antidepressants |
| Estradiol | Frequency Unknown |  | Hormones |
| Clomipramine | Frequency Unknown | Frequency Unknown | Antidepressants |
| Clozapine | Frequency Unknown |  | Antipsychotic |
| methylphenidate | Frequency Unknown |  | Hormones |
| Contraceptives | Frequency Unknown |  | Hormones |
| Cyproterone | Frequency Unknown |  | antiandrogen |
| valproate | Very common/ common |  | Mood-stabiliser |
| flupenthixol | Frequency Unknown |  | Antipsychotic |
| Depo-Provera | Frequency Unknown |  | Antipsychotic |
| Dosulepin | Frequency Unknown | Frequency Unknown | Antidepressant |
| Doxazosin | uncommon |  | alpha-1 adrenergic blockers |
| Duloxetine | Very common/ common |  | Antidepressant |
| Escitalopram | Frequency Unknown |  | Antidepressant |
| Fluoxetine | Frequency Unknown |  | Antidepressant |
| Flupentixol | Frequency Unknown |  | Antipsychotic |
| Fluphenazine Decanoate | Frequency Unknown |  | Antipsychotic |
| Gabapentin | Very common/ common |  | Anti-convulsant |
| HRT | Frequency Unknown |  | Hormones |
| Haloperidol | Frequency Unknown |  | Antipsychotic |
| Imipramine | Frequency Unknown | Frequency Unknown | Antidepressant |
| Levetiracetam | uncommon |  | Anti-epileptic |
| Lithium | Frequency Unknown |  | Mood-stabiliser |
| Lofepramine | Frequency Unknown | Frequency Unknown | Antidepressant |
| Mirtazapine | Very common/ common |  | Antidepressant |
| Modafinil | uncommon |  | Anti-sedative |
| mometasone | uncommon |  | Corticosteroid |
| testosterone | Very common/ common |  | Hormones |
| Olanzapine | Frequency Unknown |  | Antipsychotic |
| Paliperidone | Very common/ common |  | Antipsychotic |
| Paroxetine | Frequency Unknown |  | Antidepressant |
| Prednisolone | Frequency Unknown |  | Anti-asthmatic |
| Pregabalin | Very common/ common | Rare | Anti-epileptic |
| Prochlorperazine | Frequency Unknown |  | Antipsychotic |
| Promazine | Frequency Unknown |  | Antipsychotic |
| Quetiapine | Frequency Unknown |  | Antipsychotic |
| Risperidone | Frequency Unknown |  | Antipsychotic |
| Sertraline | Frequency Unknown |  | Antidepressant |
| SodiumValproate | Very common/ common |  | Mood-stabiliser |
| trifluoperazine | Frequency Unknown |  | Antipsychotic |
| Steroids | Frequency Unknown |  | Steroids |
| Sulpiride | Frequency Unknown |  | Antipsychotic |
| Terazosin | Frequency Unknown |  | Anti-hypertensive |
| Trifluoperazine | Frequency Unknown |  | Antipsychotic |
| Trimipramine | Frequency Unknown | Frequency Unknown | Antidepressant |
| Zuclopenthixol | Frequency Unknown | Frequency Unknown | Antipsychotic |

Supplementary table 3- Statistics and parameters for polygenic risk scoring

| Polygenic risk score | Best p-value threshold | Number of SNPs | P-value | R2 |
| --- | --- | --- | --- | --- |
| Body mass index | 0.21 | 23117 | 0.489 | 0.046 |
| Waist Hip Ratio | 0.02 | 3236 | 0.252 | 0.136 |
| Schizophrenia | 0.5 | 50821 | 0 | -5.611 |
| Bipolar disorder | 0.02 | 3873 | 0.352 | 0.170 |
| Insulin resistance | 0.13 | 15803 | 0.292 | 0.139 |
| Height | 0.26 | 36973 | 0.836 | 0.637 |

Supplementary table 4 - Associations of variables with genetic PCs (PRS - polygenic risk score, BMI- body mass index)

| Variable | p-value | Correlation  Value | Genetic  Principal  Component |
| --- | --- | --- | --- |
| Ethnicity | <2.2e-16 | -0.7072 | PC1 |
| Insulin resistance PRS | 5.372e-09 | 0.597 | PC1 |
| BMI PRS | <2.2e-16 | 0.8671 | PC1 |
| Schizophrenia PRS | <2.2e-16 | 0.9577 | PC1 |
| Bipolar PRS | 8.455e-07 | -0.5414 | PC1 |
| Height PRS | <2.2e-16 | -0.9001 | PC1 |
| Ethnicity group | 1.101e-05 | 0.5088 | PC2 |
| Lymphocytes | 3.174e-08 | 0.5017 | PC1 |
| Neutrophils | 2.242e-08 | -0.5065 | PC1 |

Supplementary table 5- Association of expression principal component with variables (PRS = polygenic risk score, PC = principal component)

| Variable | p-value | Correlation  Value | Expression Principal Component |
| --- | --- | --- | --- |
| Waist | 0.0007156 | -0.3207 | PC1 |
| Schizophrenia PRS | 0.000238 | 0.3466 | PC1 |
| Height PRS | 3.685e-05 | -0.386 | PC1 |
| PC1 genetic | 6.381e-05 | 0.375 | PC1 |
| Schizophrenia PRS | 0.0004618 | 0.3313 | PC17 |
| Height PRS | 0.0002469 | -0.3458 | PC17 |
| PC1 genetic | 0.0001358 | 0.359 | PC17 |
| Lymphocytes | <2.2e-16 | 0.8769 | PC1 |
| Neutrophils | <2.2e-16 | -0.8279 | PC1 |
| Monocytes | 7.457e-07 | 0.4552 | PC6 |
| Monocytes | 0.0002973 | -0.3416 | PC7 |

Supplementary table 6- association of expression modules with clinical and genetic variables

| Module | p_val | corr_val | Associated variable |
| --- | --- | --- | --- |
| Pink | 0.000654 | 0.323 | Hip circumference |
| Grey | 0.000327 | 0.339 | Height |
| Grey | 0.000454 | -0.332 | Sex |
| Green-Yellow | 0.000623 | 0.324 | Genetic PC1 |

Supplementary table 7-Demographics for the training and testing subsets for modelling

| **Variable** | **Training (n = 108)** | **Testing (n = 108)** | **Training (n = 284)** | **Testing (n = 284)** |
| --- | --- | --- | --- | --- |
| Number of samples | 82 | 26 | 214 | 70 |
| Age | 45.02 | 46.43 | 45.02 | 44.65 |
| %Male | 68.29% | 46.15% | 60.28% | 58.5% |
| Mean baseline BMI | 31.55 | 31.92 | 30.97 | 30.99 |
| Mean PANSS | 49.74 | 45.75 | 50.89 | 49.98 |
| % smokers | 65.85% | 50% | 63.5% | 60.0% |
| Mean number of weight promoting drugs | 2.37 | 2.42 | 2.065 | 2.371 |
| **Diagnosis** |  |  |  |  |
| Schizophrenia | 56 | 16 | 141 | 49 |
| Schizotypal | 1 | 0 | 1 | 0 |
| Delusional | 0 | 0 | 2 | 1 |
| Schizoaffective | 10 | 3 | 34 | 10 |
| General psychosis | 1 | 1 | 1 | 2 |
| Bipolar disorder (with psychosis) | 11 | 5 | 27 | 5 |
| Depression | 3 | 1 | 8 | 3 |
| **Ethnicity** |  |  |  |  |
| White | 43 | 14 | 121 | 38 |
| Black Caribbean | 24 | 5 | 50 | 17 |
| Black African | 5 | 3 | 18 | 6 |
| Asian | 6 | 1 | 7 | 4 |
| Mixed/Other | 4 | 3 | 18 | 5 |

Supplementary table 8- Variable importance (top 20) for the regression models (BP = blood pressure, BMI = body mass index, GAF- global assessment of functioning, PCS- physical component, IPAQ -International Physical Activity Questionnaire, GLM-generalised linear model)

| Model A (GLM) | Model B (GLM) | Model C (GLM) | Model D (GLM) | Model E (GLM) |
| --- | --- | --- | --- | --- |
| Pink module  BMI  Hip  PC10 genetic  Fried food  Waist  Diastolic BP  Weight | BMI  Hip  PC10 genetic  Fried food  Waist  Diastolic BP  Weight | BMI  Hip  Fried food  Waist  Diastolic BP  Weight | Pink module  BMI  Hip  Fried food  Waist  Diastolic BP  Weight | BMI  Waist  Hip  Height |

Supplementary table 9- Variable importance (top 20) for the classification models (BP = blood pressure, BMI = body mass index, GAF- global assessment of functioning, PCS- physical component score , IPAQ - International Physical Activity Questionnaire, HBA1C-glycated haemoglobin for assessment of average blood sugar over time , HDL-high density lipoprotein, LDL- low density lipoprotein. PANNS - positive and negative syndrome scale for assessment of psychosis, MDRAS-Montgomery-Asberg Depression Rating score, WHR-Waist-Hip ratio, PRS- polygenic risk score, MCS- mental component score, GLM-generalised linear model, KNN- k-nearest neighbours, RF - random forest)

| Model A (KNN) | Model B (KNN) | Model C (KNN) | Model D (KNN) | Model E (GLM) | Model E (RF) |
| --- | --- | --- | --- | --- | --- |
| HBA1C percentage  HBA1C measure  PC10 genetic  Fasting glucose  PC9 genetic  Green-yellow module  PCS  Weight  Magenta module  Diastolic BP  BMI  Smoker Y/N  Systolic BP  PC6 genetic  WHR  IPAQ (walk)  Cigarettes/day  Weight gain drugs  Grey module  Hip | HBA1C percentage  HBA1C measure  PC10 genetic  Fasting glucose  PC9 genetic  PCS  Weight  Diastolic BP  Smoker Y/N  BMI  Systolic BP  PC6 genetic  WHR  IPAQ (walk)  cigarettes/day  Weight gain drugs  Hip  Bipolar PRS  Walk (hrs)  PANNS total score | HBA1C percentage  HBA1C measure  Fasting glucose  PCS  Weight  Diastolic BP  Smoker Y/N  BMI  Systolic BP  WHR  IPAQ (walk)  Cigarettes/day  Weight gain drugs  Hip  Walk (hrs)  PANNS total score  MCS  Borough  Fat added to diet (baking)  Total fibre intake | HBA1C percentage  HBA1C measure  Fasting glucose  Green-yellow module  PCS  Weight  Magenta module  Diastolic BP  Smoker Y/N  BMI  Systolic BP  WHR  IPAQ (walk)  Cigarettes/day  Weight gain drugs  Grey module  Hip  Walk (hrs)  PANNS total score  MCS | Smoker Y/N  Intervention status  Sex  Blood HDL  Fried food  Ethnicity  Weight gain drugs  HBA1C percentage  GAF range  WHR  Unsaturated fat (category)  Fat added to diet (bread/veg)  Fat added to diet (baking)  Place of birth(UK/abroad)  Fasting glucose  Borough  PANSS (positive)  BMI  Diet-cereal/porridge  Blood triglycerides | GAF score  Walk (hrs)  Fasting glucose  GAF range  Systolic BP  Borough  PANNS total score  HBA1C measure  MDRAS score  Fibre (category)  Smoker (Y/N)  Fasting glucose  Total exercise (walking)  PCS  PANSS- gpp  Waist  Diet-cereal/porridge  Blood HDL  Fried Food  IPAQ (walk)  Intervention status |

Supplementary table 10-Summary of results for all regression models tested. Measures : RMSE= Root Mean Squared Error, R2 = R-squared, correlation indicates the agreement between predicted and actual values for the test data. Models: RegularRidge = Ridge regression. boostedLM = boosted linear models. Glmnet = Generalised Linear Models. Elastic net = elastic net. ICR = Independent Component Regression. svmLinear = Support Vector machine model with linear kernel. Knn = K-nearest neighbours. Cart = Classification and Regression Trees. Bagging = tree models with bagged method. Rf = Random forest. Gbm = generalised boosting model

| A | Model A Train RMSE | Model A Train R2 | Model A correlation | Model A Test RMSE | Model A Test R2 |
| --- | --- | --- | --- | --- | --- |
| **RegularRidge** | 4.54 | 0.726 | 0.781 | 4.925 | 0.443 |
| **boostedLM** | 6.76 | 0.297 | 0.331 | 6.568 | 0.009 |
| **Glmnet** | 3.48 | 0.827 | 0.896 | 3.035 | 0.788 |
| **elasticnet** | 3.67 | 0.803 | 0.900 | 2.946 | 0.801 |
| **ICR** | 4.94 | 0.616 | 0.824 | 3.833 | 0.663 |
| **SVMlinear** | 5.93 | 0.565 | 0.716 | 6.142 | 0.134 |
| **knn** | 4.27 | 0.748 | 0.721 | 4.637 | 0.506 |
| **cart** | 4.02 | 0.756 | 0.857 | 4.211 | 0.593 |
| **bagging** | 3.63 | 0.813 | 0.895 | 3.113 | 0.777 |
| **rf** | 3.57 | 0.823 | 0.896 | 3.020 | 0.791 |
| **gbm** | 4.02 | 0.771 | 0.872 | 3.252 | 0.757 |
| B | Model B Train RMSE | Model B Train R2 | Model B correlation | Model B Test RMSE | Model B Test R2 |
| **RegularRidge** | 4.43 | 0.733 | 0.834 | 4.496 | 0.536 |
| **boostedLM** | 6.68 | 0.337 | 0.772 | 5.184 | 0.383 |
| **Glmnet** | 3.45 | 0.83 | 0.900 | 2.981 | 0.796 |
| **elasticnet** | 3.55 | 0.83 | 0.910 | 2.981 | 0.796 |
| **ICR** | 5.07 | 0.582 | 0.833 | 3.773 | 0.673 |
| **SVMlinear** | 5.25 | 0.638 | 0.773 | 5.788 | 0.231 |
| **knn** | 4.27 | 0.748 | 0.721 | 4.637 | 0.506 |
| **cart** | 4.02 | 0.756 | 0.857 | 4.211 | 0.593 |
| **bagging** | 3.61 | 0.815 | 0.895 | 3.115 | 0.777 |
| **rf** | 3.6 | 0.824 | 0.889 | 3.169 | 0.769 |
| **gbm** | 3.95 | 0.78 | 0.886 | 3.090 | 0.781 |
| C | Model C Train RMSE | Model C Train R2 | Model C correlation | Model C Test RMSE | Model C Test R2 |
| **RegularRidge** | 4.16 | 0.763 | 0.885 | 3.540 | 0.712 |
| **boostedLM** | 6.41 | 0.428 | 0.769 | 5.225 | 0.373 |
| **Glmnet** | 3.43 | 0.832 | 0.900 | 2.981 | 0.796 |
| **elasticnet** | 3.66 | 0.805 | 0.886 | 3.191 | 0.766 |
| **ICR** | 4.37 | 0.695 | 0.859 | 3.621 | 0.699 |
| **SVMlinear** | 4.55 | 0.709 | 0.870 | 3.756 | 0.676 |
| **knn** | 4.27 | 0.748 | 0.721 | 4.637 | 0.506 |
| **cart** | 4.02 | 0.756 | 0.857 | 4.211 | 0.593 |
| **bagging** | 3.59 | 0.816 | 0.893 | 3.129 | 0.775 |
| **rf** | 3.57 | 0.823 | 0.896 | 3.069 | 0.784 |
| **gbm** | 3.87 | 0.789 | 0.879 | 3.203 | 0.764 |
| D | Model D Train RMSE | Model D Train R2 | Model D correlation | Model D Test RMSE | Model D Test R2 |
| **RegularRidge** | 4.56 | 0.729 | 0.839 | 3.976 | 0.637 |
| **boostedLM** | 6.47 | 0.368 | 0.313 | 6.525 | 0.022 |
| **Glmnet** | 3.46 | 0.829 | 0.896 | 3.035 | 0.788 |
| **elasticnet** | 3.81 | 0.82 | 0.912 | 3.840 | 0.661 |
| **ICR** | 4.48 | 0.687 | 0.852 | 3.690 | 0.687 |
| **SVMlinear** | 6.04 | 0.567 | 0.817 | 4.070 | 0.620 |
| **knn** | 4.27 | 0.748 | 0.721 | 4.637 | 0.506 |
| **cart** | 4.02 | 0.756 | 0.857 | 4.211 | 0.593 |
| **bagging** | 3.62 | 0.813 | 0.894 | 3.121 | 0.776 |
| **rf** | 3.54 | 0.822 | 0.906 | 2.904 | 0.806 |
| **gbm** | 3.95 | 0.778 | 0.868 | 3.300 | 0.750 |
| E | Model E Train RMSE | Model E Train R2 | Model E correlation | Model E Test RMSE | Model E Test R2 |
| **RegularRidge** | 3.730 | 0.750 | 0.905 | 3.066 | 0.800 |
| **boostedLM** | 5.790 | 0.588 | 0.726 | 5.634 | 0.326 |
| **Glmnet** | 3.510 | 0.782 | 0.919 | 2.840 | 0.829 |
| **elasticnet** | 3.470 | 0.777 | 0.922 | 2.695 | 0.846 |
| **ICR** | 4.900 | 0.547 | 0.728 | 4.721 | 0.526 |
| **SVMlinear** | 3.640 | 0.757 | 0.897 | 3.131 | 0.792 |
| **knn** | 4.490 | 0.633 | 0.805 | 4.172 | 0.630 |
| **cart** | 4.180 | 0.670 | 0.819 | 3.954 | 0.668 |
| **bagging** | 3.410 | 0.779 | 0.868 | 3.495 | 0.741 |
| **rf** | 3.350 | 0.789 | 0.859 | 3.541 | 0.734 |
| **gbm** | 3.400 | 0.782 | 0.878 | 3.320 | 0.766 |

Supplementary table 11- Summary of all classification models tested. Measures : NPV= Negative Predictive Value , PPV= positive predictive value. Models: Glmnet = Generalised Linear Models, svmRad= Support Vector machine model with radial kernel., svmPoly = Support Vector machine model with polynomial kernel, svmLinear = Support Vector machine model with linear kernel, Knn = K-nearest neighbours. Cart = Classification and Regression Trees., c50 -classification trees with Quinlan's C5.0 algorithm , Bagging = tree models with bagged method. Rf = Random forest. Gbm = generalised boosting model

| A | Model A  Train Accuracy | Model A train Kappa | Model A Test Accuracy | Model A Test Kappa | Model A Test Sensitivity | Model A Test Specificity | Model A Test PPV | Model A Test NPV |
| --- | --- | --- | --- | --- | --- | --- | --- | --- |
| **glmnet** | 0.471 | -0.105 | 0.731 | 0.442 | 0.750 | 0.700 | 0.800 | 0.636 |
| **svmRad** | 0.586 | 0 | 0.577 | 0.000 | 0.577 | NA | NA | NA |
| **svmPoly** | 0.586 | 0 | 0.577 | 0.000 | 0.577 | NA | NA | NA |
| **svmLinear** | 0.497 | -0.0513 | 0.654 | 0.282 | 0.688 | 0.600 | 0.733 | 0.545 |
| **knn** | 0.591 | 0.0964 | 0.577 | 0.077 | 0.600 | 0.500 | 0.800 | 0.273 |
| **cart** | 0.413 | -0.253 | 0.577 | 0.000 | 0.577 | NA | NA | NA |
| **c50** | 0.557 | 0.0811 | 0.500 | -0.063 | 0.556 | 0.375 | 0.667 | 0.273 |
| **bagging** | 0.463 | -0.15 | 0.500 | -0.037 | 0.563 | 0.400 | 0.600 | 0.364 |
| **rf** | 0.537 | -0.0651 | 0.615 | 0.103 | 0.600 | 1.000 | 1.000 | 0.091 |
| **gbm** | 0.486 | -0.0958 | 0.577 | 0.123 | 0.625 | 0.500 | 0.667 | 0.455 |
| B | Model B Train Accuracy | Model B train Kappa | Model B Test Accuracy | Model B Test Kappa | Model B Test Sensitivity | Model B Test Specificity | Model B Test PPV | Model B Test NPV |
| **glmnet** | 0.537 | 0.0495 | 0.577 | 0.101 | 0.611 | 0.500 | 0.733 | 0.364 |
| **svmRad** | 0.586 | 0 | 0.577 | 0.000 | 0.577 | NA | NA | NA |
| **svmPoly** | 0.586 | 0 | 0.577 | 0.000 | 0.577 | NA | NA | NA |
| **svmLinear** | 0.541 | 0.0562 | 0.577 | 0.123 | 0.625 | 0.500 | 0.667 | 0.455 |
| **knn** | 0.591 | 0.0964 | 0.577 | 0.077 | 0.600 | 0.500 | 0.800 | 0.273 |
| **cart** | 0.433 | -0.186 | 0.615 | 0.172 | 0.632 | 0.571 | 0.800 | 0.364 |
| **c50** | 0.574 | 0.117 | 0.500 | -0.063 | 0.556 | 0.375 | 0.667 | 0.273 |
| **bagging** | 0.472 | -0.121 | 0.577 | 0.101 | 0.611 | 0.500 | 0.733 | 0.364 |
| **rf** | 0.545 | -0.0382 | 0.615 | 0.103 | 0.600 | 1.000 | 1.000 | 0.091 |
| **gbm** | 0.514 | -0.0336 | 0.577 | 0.123 | 0.625 | 0.500 | 0.667 | 0.455 |
| C | Model C Train Accuracy | Model C train Kappa | Model C Test Accuracy | Model C Test Kappa | Model C Test Sensitivity | Model C Test Specificity | Model C Test PPV | Model C Test NPV |
| **glmnet** | 0.538 | 0.0554 | 0.615 | 0.193 | 0.647 | 0.556 | 0.733 | 0.455 |
| **svmRad** | 0.586 | 0 | 0.577 | 0.000 | 0.577 | NA | NA | NA |
| **svmPoly** | 0.586 | 0 | 0.577 | 0.000 | 0.577 | NA | NA | NA |
| **svmLinear** | 0.518 | 0.0171 | 0.577 | 0.123 | 0.625 | 0.500 | 0.667 | 0.455 |
| **knn** | 0.591 | 0.0964 | 0.577 | 0.077 | 0.600 | 0.500 | 0.800 | 0.273 |
| **cart** | 0.493 | -0.0348 | 0.615 | 0.193 | 0.647 | 0.556 | 0.733 | 0.455 |
| **c50** | 0.565 | 0.0942 | 0.385 | -0.325 | 0.474 | 0.143 | 0.600 | 0.091 |
| **bagging** | 0.515 | -0.0157 | 0.615 | 0.193 | 0.647 | 0.556 | 0.733 | 0.455 |
| **rf** | 0.559 | 0.00888 | 0.577 | 0.053 | 0.591 | 0.500 | 0.867 | 0.182 |
| **gbm** | 0.555 | 0.0699 | 0.577 | 0.101 | 0.611 | 0.500 | 0.733 | 0.364 |
| D | Model D Train Accuracy | Model D train Kappa | Model D Test Accuracy | Model D Test Kappa | Model D Test Sensitivity | Model D Test Specificity | Model D Test PPV | Model D Test NPV |
| **glmnet** | 0.547 | 0.0586 | 0.769 | 0.527 | 0.800 | 0.727 | 0.800 | 0.727 |
| **svmRad** | 0.586 | 0 | 0.577 | 0.000 | 0.577 | NA | NA | NA |
| **svmPoly** | 0.586 | 0 | 0.577 | 0.000 | 0.577 | NA | NA | NA |
| **svmLinear** | 0.535 | 0.0379 | 0.692 | 0.370 | 0.733 | 0.636 | 0.733 | 0.636 |
| **knn** | 0.591 | 0.0964 | 0.577 | 0.077 | 0.600 | 0.500 | 0.800 | 0.273 |
| **cart** | 0.444 | -0.19 | 0.577 | 0.000 | 0.577 | NA | NA | NA |
| **c50** | 0.538 | 0.0462 | 0.385 | -0.325 | 0.474 | 0.143 | 0.600 | 0.091 |
| **bagging** | 0.481 | -0.103 | 0.538 | 0.031 | 0.588 | 0.444 | 0.667 | 0.364 |
| **rf** | 0.556 | -0.0143 | 0.577 | 0.053 | 0.591 | 0.500 | 0.867 | 0.182 |
| **gbm** | 0.521 | -0.022 | 0.538 | 0.006 | 0.579 | 0.429 | 0.733 | 0.273 |
| E | Model E Train Accuracy | Model E train Kappa | Model E Test Accuracy | Model E Test Kappa | Model E Test Sensitivity | Model E Test Specificity | Model E Test PPV | Model E Test NPV |
| **glmnet** | 0.574 | 0.056 | 0.600 | 0.132 | 0.660 | 0.478 | 0.721 | 0.407 |
| **svmRad** | 0.617 | 0.000 | 0.614 | 0.000 | 0.614 | NA | NA | NA |
| **svmPoly** | 0.617 | 0.000 | 0.614 | 0.000 | 0.614 | NA | NA | NA |
| **svmLinear** | 0.556 | 0.038 | 0.629 | 0.182 | 0.673 | 0.524 | 0.767 | 0.407 |
| **knn** | 0.570 | 0.023 | 0.571 | 0.083 | 0.644 | 0.440 | 0.674 | 0.407 |
| **cart** | 0.541 | 0.006 | 0.571 | 0.057 | 0.633 | 0.429 | 0.721 | 0.333 |
| **c50** | 0.566 | 0.034 | 0.571 | 0.043 | 0.627 | 0.421 | 0.744 | 0.296 |
| **bagging** | 0.573 | 0.039 | 0.600 | 0.093 | 0.642 | 0.471 | 0.791 | 0.296 |
| **rf** | 0.608 | 0.052 | 0.586 | -0.022 | 0.609 | 0.333 | 0.907 | 0.074 |
| **gbm** | 0.591 | 0.068 | 0.543 | -0.068 | 0.596 | 0.308 | 0.791 | 0.148 |

Supplementary table 12-Summary of regression modelling results for genetic and expression data alone. Measures : RMSE= Root Mean Squared Error, R2 = R-squared, correlation indicates the agreement between predicted and actual values for the test data. Models: RegularRidge = Ridge regression. boostedLM = boosted linear models. Glmnet = Generalised Linear Models. Elastic net = elastic net. ICR = Independent Component Regression. svmLinear = Support Vector machine model with linear kernel. Knn = K-nearest neighbours. Cart = Classification and Regression Trees. Bagging = tree models with bagged method. Rf = Random forest. Gbm = generalised boosting model

| Genes model ( n= 108) | Genes model  Train RMSE | Genes model  Train R2 | Genes model  Correlation | Genes model  Test RMSE | Genes model  Test R2 |
| --- | --- | --- | --- | --- | --- |
| **RegularRidge** | 8.05 | 0.126 | 0.09047 | 6.985 | -0.1207 |
| **boostedLM** | 7.48 | 0.149 | 0.07841 | 6.646 | -0.01442 |
| **Glmnet** | 7.76 | 0.140 | 0.1147 | 6.684 | -0.02606 |
| **elasticnet** | 7.36 | 0.121 | 0.1887 | 6.57 | 0.008493 |
| **ICR** | 7.39 | 0.0881 | 0.1965 | 6.587 | 0.003216 |
| **SVMlinear** | 8.18 | 0.115 | 0.1947 | 6.647 | -0.01474 |
| **knn** | 7.57 | 0.136 | 0.1147 | 6.845 | -0.07604 |
| **cart** | 8.83 | 0.123 | NA | 6.614 | -0.004735 |
| **bagging** | 7.82 | 0.143 | 0.2012 | 6.506 | 0.02794 |
| **rf** | 7.73 | 0.141 | 0.3492 | 6.324 | 0.0814 |
| **gbm** | 7.83 | 0.144 | -0.3691 | 7.749 | -0.379 |
| Genes model ( n= 186) | Genes model  Train RMSE | Genes model  Train R2 | Genes model  Correlation | Genes model  Test RMSE | Genes model  Test R2 |
| **RegularRidge** | 7.59 | 0.1090 | 0.3399 | 7.633 | 0.1117 |
| **boostedLM** | 7.32 | 0.1070 | 0.3281 | 7.755 | 0.08303 |
| **Glmnet** | 7.42 | 0.1050 | 0.3137 | 7.743 | 0.08581 |
| **elasticnet** | 7.26 | 0.0943 | 0.2453 | 8.01 | 0.02171 |
| **ICR** | 7.29 | 0.0711 | -0.12 | 8.145 | -0.01 |
| **SVMlinear** | 7.54 | 0.1070 | 0.3257 | 7.777 | 0.07773 |
| **knn** | 7.47 | 0.0727 | -0.009263 | 8.565 | -0.1186 |
| **cart** | 7.81 | 0.0390 | NA | 8.117 | -0.00469 |
| **bagging** | 7.41 | 0.0762 | 0.3016 | 7.737 | 0.08717 |
| **rf** | 7.19 | 0.0970 | 0.3242 | 7.725 | 0.09004 |
| **gbm** | 7.38 | 0.0750 | 0.2008 | 7.99 | 0.02655 |
| Expression model | Expression model  Train RMSE | Expression model  Train R2 | Expression model  Correlation | Expression model  Test RMSE | Expression model  Test R2 |
| **RegularRidge** | 7.35 | 0.219 | -0.1048 | 8.49 | -0.6555 |
| **boostedLM** | 6.86 | 0.272 | -0.09713 | 7.879 | -0.4259 |
| **Glmnet** | 6.86 | 0.279 | -0.09134 | 7.664 | -0.3489 |
| **elasticnet** | 6.90 | 0.266 | -0.09773 | 7.864 | -0.4205 |
| **ICR** | 7.35 | 0.124 | 0.06715 | 6.603 | -0.00163 |
| **SVMlinear** | 7.43 | 0.203 | -0.1088 | 8.853 | -0.7999 |
| **knn** | 7.25 | 0.173 | 0.1603 | 6.686 | -0.02682 |
| **cart** | 6.71 | 0.319 | 0.01044 | 7.982 | -0.4634 |
| **bagging** | 6.76 | 0.283 | -0.06261 | 8.066 | -0.4941 |
| **rf** | 6.97 | 0.217 | 0.02096 | 7.8 | -0.3973 |
| **gbm** | 7.08 | 0.227 | -0.1502 | 7.97 | -0.4588 |

Supplementary table 13-Summary of classification modelling results for genetic and expression data alone. Measures : : NPV= Negative Predictive Value , PPV= positive predictive value. Models: Glmnet = Generalised Linear Models, svmRad= Support Vector machine model with radial kernel., svmPoly = Support Vector machine model with polynomial kernel, svmLinear = Support Vector machine model with linear kernel, Knn = K-nearest neighbours. Cart = Classification and Regression Trees., c50 -classification trees with Quinlan's C5.0 algorithm , Bagging = tree models with bagged method. Rf = Random forest. Gbm = generalised boosting model

| Method for genes model ( n = 108) | genes model  Train Accuracy | genes model  train Kappa | genes model  Test Accuracy | genes model  Test Kappa | genes model  Test Sensitivity | genes model  Test Specificity | genes model  Test PPV | genes model  Test NPV |
| --- | --- | --- | --- | --- | --- | --- | --- | --- |
| **glmnet** | 0.551 | 0.0743 | 0.577 | 0.101 | 0.611 | 0.500 | 0.733 | 0.364 |
| **svmRad** | 0.586 | 0.0000 | 0.577 | 0.000 | 0.577 | NA | NA | NA |
| **svmPoly** | 0.591 | 0.0142 | 0.577 | 0.000 | 0.577 | NA | NA | NA |
| **svmLinear** | 0.573 | 0.0789 | 0.654 | 0.245 | 0.650 | 0.667 | 0.867 | 0.364 |
| **knn** | 0.588 | 0.1040 | 0.615 | 0.212 | 0.667 | 0.545 | 0.667 | 0.545 |
| **cart** | 0.488 | -0.1090 | 0.577 | 0.000 | 0.577 | NA | NA | NA |
| **c50** | 0.578 | -0.0113 | 0.577 | 0.000 | 0.577 | NA | NA | NA |
| **bagging** | 0.503 | -0.0412 | 0.385 | -0.292 | 0.471 | 0.222 | 0.533 | 0.182 |
| **rf** | 0.524 | -0.0422 | 0.462 | -0.103 | 0.533 | 0.364 | 0.533 | 0.364 |
| **gbm** | 0.476 | -0.1100 | 0.462 | -0.130 | 0.529 | 0.333 | 0.600 | 0.273 |
| Method for genes model ( n = 186) | genes model  Train Accuracy | genes model  train Kappa | genes model  Test Accuracy | genes model  Test Kappa | genes model  Test Sensitivity | genes model  Test Specificity | genes model  Test PPV | genes model  Test NPV |
| **glmnet** | 0.592 | 0.11500 | 0.478 | -0.165 | 0.559 | 0.250 | 0.679 | 0.167 |
| **svmRad** | 0.607 | 0.03370 | 0.543 | -0.126 | 0.581 | 0.000 | 0.893 | 0.000 |
| **svmPoly** | 0.602 | 0.00604 | 0.609 | 0.000 | 0.609 | NA | NA | NA |
| **svmLinear** | 0.598 | 0.10300 | 0.522 | -0.115 | 0.579 | 0.250 | 0.786 | 0.111 |
| **knn** | 0.550 | 0.03600 | 0.478 | -0.117 | 0.567 | 0.313 | 0.607 | 0.278 |
| **cart** | 0.515 | 0.13400 | 0.609 | 0.000 | 0.609 | NA | NA | NA |
| **c50** | 0.600 | 0.00000 | 0.609 | 0.000 | 0.609 | NA | NA | NA |
| **bagging** | 0.519 | 0.03670 | 0.522 | -0.091 | 0.583 | 0.300 | 0.750 | 0.167 |
| **rf** | 0.551 | 0.02630 | 0.565 | -0.036 | 0.600 | 0.333 | 0.857 | 0.111 |
| **gbm** | 0.534 | 0.03860 | 0.652 | 0.207 | 0.667 | 0.600 | 0.857 | 0.333 |
| Method for Expression model | Expression model  Train Accuracy | Expression model  train Kappa | Expression model  Test Accuracy | Expression model  Test Kappa | Expression model  Test Sensitivity | Expression model  Test Specificity | Expression model  Test PPV | Expression model  Test NPV |
| **glmnet** | 0.503 | -0.08870 | 0.577 | 0.123 | 0.625 | 0.500 | 0.667 | 0.455 |
| **svmRad** | 0.586 | 0.00000 | 0.577 | 0.000 | 0.577 | NA | NA | NA |
| **svmPoly** | 0.586 | 0.00000 | 0.577 | 0.000 | 0.577 | NA | NA | NA |
| **svmLinear** | 0.514 | -0.04950 | 0.500 | -0.037 | 0.563 | 0.400 | 0.600 | 0.364 |
| **knn** | 0.530 | -0.00843 | 0.500 | -0.090 | 0.550 | 0.333 | 0.733 | 0.182 |
| **cart** | 0.527 | -0.09330 | 0.577 | 0.000 | 0.577 | NA | NA | NA |
| **c50** | 0.574 | -0.02350 | 0.577 | 0.000 | 0.577 | NA | NA | NA |
| **bagging** | 0.463 | -0.14900 | 0.577 | 0.101 | 0.611 | 0.500 | 0.733 | 0.364 |
| **rf** | 0.484 | -0.13700 | 0.577 | 0.101 | 0.611 | 0.500 | 0.733 | 0.364 |
| **gbm** | 0.500 | -0.07040 | 0.615 | 0.193 | 0.647 | 0.556 | 0.733" | 0.4545 |

Supplementary table 14-Module gene enrichment, assessed via PANTHER and AMIGO

| Module | Number of probes | Pathway enrichment | Fold enrichment | Bonferroni corrected p-value | Annotation dataset utilised |
| --- | --- | --- | --- | --- | --- |
| Black | 178 | NA | NA | NA | All tested |
| Blue | 1034 | Respiratory electron transport, ATP synthesis by chemiosmotic couling and heat production by uncoupling proteins | 2.29 | 0.033 | Reactome Pathways |
| Brown | 693 | Interferon alpha-beta signalling | 4.87 | 3.36^-7^ | Reactome Pathways |
| Brown | 693 | Innate Immune system | 1.62 | 0.0186 | Reactome Pathways |
| Brown | 693 | Interferon gamma signalling | 3.67 | 4.42^-5^ | Reactome Pathways |
| Brown | 693 | Type 1 interferon signalling pathway | 5.17 | 2.4^-8^ | GO biological process |
| Brown | 693 | Negative regulation of viral replication | 4.16 | 0.0305 | GO biological process |
| Brown | 693 | Interferon gamma mediated signalling pathway | 3.9 | 4.7^-5^ | GO biological process |
| Brown | 693 | Defense response to virus | 2.96 | 5.7^-5^ | GO biological process |
| Brown | 693 | Activation of innate immune response | 2.17 | 0.028 | GO biological process |
| Brown | 693 | Regulation of cytokine production | 1.93 | 5.79-4 | GO biological process |
| Brown | 693 | Cellular defense response | 2.92 | 3.36^-5^ | PANTHER GO-SLIM Biological process |
| Brown | 693 | Embryo development | 2.78 | 0.047 | PANTHER GO-SLIM Biological process |
| Brown | 693 | Nervous System development | 2.02 | 0.015 | PANTHER GO-SLIM Biological process |
| Brown | 693 | Immune response | 1.75 | 0.019 | PANTHER GO-SLIM Biological process |
| Brown | 693 | Intracellular signal transduction | 1.58 | 0.028 | PANTHER GO-SLIM Biological process |
| Green | 228 | Ubiquitin ligase complex | 3.10 | 0.029 | GO cellular component complete |
| Green-yellow | 41 | NA | NA | NA | All tested |
| Grey | 1680 | NA | NA | NA | All tested |
| Magenta | 160 | Cell-cell junction | 3.43 | 0.038 | GO cellular component complete |
| Magenta | 160 | Cell-extracellular matix interactions | 14.46 | 0.046 | Reactome pathways |
| Magenta | 160 | Gene expression | 0.40 | 0.0031 | GO biological process complete |
| Magenta | 160 | RNA metabolic process | 0.35 | 0.0025 | GO biological process complete |
| Magenta | 160 | Nucleic acid binding | 0.43 | 0.0018 | GO molecular function complete |
| Magenta | 160 | Cytoskeletal protein | 2.89 | 0.0043 | PANTHER protein class |
| Magenta | 160 | Structural constituent of cytoskeleton | 3.69 | 0.00038 | PANTHER GO-Slim Molecular function |
| Pink | 166 | Signal transduction | 1.87 | 0.024 | PANTHER GO-Slim Biological process |
| Pink | 166 | External side of plasma membrane | 4.14 | 0.0091 | GO cellular component complete |
| Pink | 166 | Intracellular organelle part | 0.76 | 0.049 | GO cellular component complete |
| Purple | 117 | NA | NA | NA | All tested |
| Red | 194 | Structural component of ribosome | 3.30 | 0.018 | PANTHER GO-Slim Molecular function |
| Red | 194 | Cytosolic ribosome | 4.13 | 0.020 | GO cellular component complete |
| Red | 194 | Ribosomal subunit | 3.37 | 0.048 | GO cellular component complete |
| Turquoise | 1280 | NA | NA | NA | All tested |
| Yellow | 588 | ribosome | 2.54 | 0.0069 | PANTHER GO-SLIM Cellular component |
| Yellow | 588 | cytosol | 1.93 | 0.021 | PANTHER GO-SLIM Cellular component |
| Yellow | 588 | Nucleic Acid binding | 1.44 | 0.0179 | PANTHER Protein class |
| Yellow | 588 | RNA binding | 1.59 | 0.0005 | GO Molecular function complete |
| Yellow | 588 | Viral transcription | 3.44 | 0.00027 | GO biological process complete |
| Yellow | 588 | SRP dependent cotranslational protein targeting to membrane | 3.17 | .0104 | GO biological process complete |
| Yellow | 588 | Nuclear transcribed mRNA catabolic process, nonsense medicated decay | 2.99 | 0.0103 | GO biological process complete |
| Yellow | 588 | Translational initiation | 2.71 | 0.024 | GO biological process complete |
| Yellow | 588 | RNA processing | 1.82 | 0.011 | GO biological process complete |
| Yellow | 588 | Cytosolic ribosome | 2.77 | 0.024 | GO cellular component complete |
| Yellow | 588 | Chromosomal part | 1.81 | 0.0459 | GO cellular component complete |
| Yellow | 588 | nucleoplasm | 1.43 | 7.7^-5^ | GO cellular component complete |
| Yellow | 588 | Formation of free 40S subunits | 3.42 | 0.000152 | Reactome pathways |
| Yellow | 588 | Selenocysteine synthesis | 3.4 | 0.000899 | Reactome pathways |
| Yellow | 588 | L13a-mediated translational silencing of Ceruloplasmin expression | 3.3 | 0.000178 | Reactome pathways |
| Yellow | 588 | Viral mRNA transl;ation | 3.26 | 0.00321 | Reactome pathways |
| Yellow | 588 | Peptide chain elongation | 3.26 | 0.00321 | Reactome pathways |
| Yellow | 588 | Nonsense Mediated Decay independent of Exon junction Complex | 3.20 | 0.00247 | Reactome pathways |
| Yellow | 588 | Eukaryotic translation termination | 3.18 | 0.0047 | Reactome pathways |
| Yellow | 588 | GTP hydrolysis and joining of 60S ribosomal subunit | 3.14 | 0.000748 | Reactome pathways |
| Yellow | 588 | SRP dependent cotranslational protein targeting to membrane | 3.08 | 0.00108 | Reactome pathways |
| Yellow | 588 | Nonsense Mediated Decay enhanced by Exon junction Complex | 2.93 | 0.00662 | Reactome pathways |

## Supplementary methods

### Study and participant details

IMPACT investigated effectiveness of cognitive behavioural therapy (CBT), motivational interviews, and health promotion at improving health behaviours in a community setting. The control group of patients had treatment as usual. Participants were in their usual community treatment setting, excluding specialist first episode psychosis teams. They were excluded if they had primary diagnosis of learning disability, co-existing physical health problems that would independently impact on metabolic measures and/or substance use habits, were pregnant/less than 6 months post-partum or had life threatening/terminal medical conditions requiring intensive care*.*

## Supplementary figures:


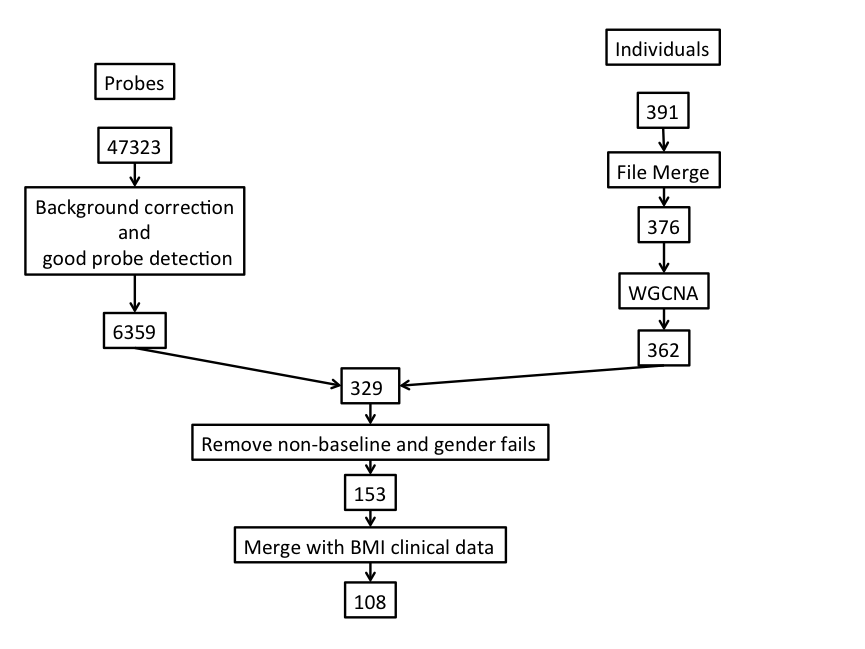


Supplementary figure 1- Outline of main steps of expression data quality control
